# Assessment of Ethical Ideals and Ethical Manners in Care of Older People

**DOI:** 10.1155/2013/374132

**Published:** 2013-03-13

**Authors:** Marianne Frilund, Lisbeth Fagerström, Katie Eriksson, Patrik Eklund

**Affiliations:** ^1^Åbo Akademi University, Vaasa, Finland; ^2^Novia University of Applied Sciences, Vaasa, Finland; ^3^Buskerud University College, Drammen, Norway; ^4^Department of Caring Science, Åbo Akademi University, Vaasa, Finland; ^5^Helsinki Hospital District of Helsinki and Uusimaa, Finland; ^6^Department of Computing Science, Umeå University, Umeå, Sweden

## Abstract

The aim of this study is to establish structured clusters and well-defined ontological entities (nodes) describing ethical values as both ideal and opportunity for ethical manner as perceived by thecaregiver.In this study, we use Bayesian Belief Networks (BBNs) to analyse ethical values (ethos) and ethical manners in daily work with older people. Material is based on questionnaire data collected by the instrument for the self-assessment of individual ethos in the care of older people(ISAEC) in spring 2007 in a municipality in Western Finland. This study is unique in its kind, both concerning the selected approach and methodological questions. BBNs have not been used significantly in nursing research, nor are there any studies that examine the ethical possibilities with focus on the probable effects upon changing conditions.

## 1. Introduction

Ethical discussions between caregivers affect the quality of the older person's care, and Ågren Bolmsjö et al. [1] have found that ethical decision-making supports ethically good care of patients. Berggren et al. [2] associate the discussion of ethical values with a deeper level of communication, and in order to achieve depth in such a dialogue, an ethical code and a set of ethical values which penetrate caring are needed. Awareness of such ethical values equips caregivers with a freedom and strength to make conscious decisions to do well and to do right in a given care situation. A caregiver's ability to do well and do right is strengthened in the dialogue between caregivers and other health care professionals [3].

In this study, we use Bayesian Belief Networks [4, 5] (BBNs) to analyse ethical values (ethos) and ethical manners in daily work with older people. The advantage with BBNs is the possibility to use and compute with symbolic (symbolic data has no per se measurable or comparable values), as opposed to numeric or nominal (… 1,2,3,4,5 are nominal not to be seen as numerical …), data. Linear regression and comparable methods require numeric data for its computations. Data used in this study are nominal in the answers to questions in the questionnaire, but inherently symbolic when arriving at ethical data and classifications of ethical manner. Further, BBNs are able to manage stochasticity and uncertainty and can work simultaneously with objective and subjective probabilities in one and the same model.

Material is based on questionnaire data collected by the instrument for the self-assessment of individual ethos in the care of older people (ISAEC) in spring 2007 in a municipality in Western Finland [6]. The study is based on a caring science perspective, and caregivers' ethical values and ethical manner which are evaluated in the study have been interpreted to the theory of caritative caring ethics [7] and to previous research on ethics in the care of older people [8–13]. The caring science perspective appears in the statements of the questionnaire, and in the concept, which are given the clusters and nodes, generated with BBN.

Biostatistics or, generally speaking, statistics as used in the care domain is indeed strictly statistics. ‘‘Statistic inference” is not logic inference but ad hoc conclusions derived from statistical observations and analysis. Such conclusions are not expressed in any logical language but still within the statistical machinery. However, health and social care involving observations, assessments, and decision-making mean that somewhere along the line *statistics moves over to logics*.

Logical entailment ⊢ is a relation between premises and conclusions. It is syntactic reasoning as related to its semantic counterpart, namely, logical satisfaction ⊨. We will illuminate our epistemology with syntactic entailments, where the choice of a specific logic, first-order or otherwise, is not relevant as we are providing a complete ethical ontology in this paper.

### 1.1. Theoretical Framework

In the theory of caritative caring ethics [14] and in the previous research on ethics in caring, related to the care of older people can we see, among other, values as dignity, [10, 15–17] integrity, [11, 16, 18–20] autonomy and participation, [10, 16, 17, 21], respect and safety [13, 20, 22, 23]. We can also find different explanations about caregivers' possibilities to act in an ethical manner in the daily work with the older persons. It is not self-evident that ethical values of the caregivers turn into ethical manners in the daily work, and we have to state that *a good intention goes wrong* [11], and the caregiver encounters different ethical problems and challenges that need to be resolved. Often there is not one solution to the problem, rather, many different solutions. The essence of caring is to alleviate the patient's suffering and promote health and wellbeing. Eriksson [24] described caring ethics in terms like love and mercy, caring relationship, human dignity and respect, which accordingly affects human beings' decisions and choices in a specific manner [7, 24–26]. The *caring communion* is the deepest motive for every kind of caring. A professional caring relationship implies a responsibility of caregiver vis-à-vis the patient he/she takes care of.

An ethically aware caregiver strives to invite the patient into a caring relation that mediates strength as well as respect for the integrity and wholeness of the human being [15]. An ethically aware caregiver also strives to “do well,” “do right,” and “take responsibility,” and he/she wanted to show the patient respect [9].

To act ethically in an ontological sense is not always related to time. Acting ethically exists in the moment when goodness becomes a conscious choice for the caregiver. To act ethically in the daily work requires a professional freedom, enabling caregiver to choose and decide just in the moment when caregiver and patient meet each other. This kind of freedom goes behind routines and stereotypical behaviours and thereby promotes unique meetings.

### 1.2. Aim

The aim of this study is to establish structured clusters and well-defined ontological entities (nodes) describing ethical values as both ideal and opportunity for ethical manner as perceived by the caregiver. This additionally provides an enlargement and enrichment of the underlying ethical assumptions about ethical values and the dynamics in spectra of caregiver ethical manner. An additional objective is to evaluate the effect of fixing nodes to certain assessments levels, in order to see how other nodes are affected in themselves and from the viewpoint of the entire cluster. This in turn contributes to knowledge elicitation and epistemological enhancement with respect to the ontological framework.

*This paper focuses on the following questions:*

1. (ontological question) which are the main patterns involving ethical value and ethical manner emerging from this study, given the underlying structural entities and dynamical ethical values and manners?
2. (complementary epistemological question) which are the various types of conditional changes of ethical values and their related ethical manners that appear when fixing nodes to particular values and thereby clusters to specific characters?
3. (societal impact) how will this elicited knowledge in the end affect daily care of older people and as viewed from an ethical perspective?

## 2. Method

### 2.1. Participants

Caregivers from 10 units in the care of older persons were invited to participate in the study. Three units represented Home Care, four units Nursing Home Care, and three units Long-Term Care. A majority (*n* = 80) of the informants worked within Nursing Home Care or within Long-Term Care, whereas the remaining informants (*n* = 25) worked within Home Care.

A majority of the informants had a vocational degree, for instance, registered nurses and practical nurses. Totally, 24 caregivers had attended shorter courses according to older educational programs, such as courses for care assistants, and six of the informants lacked formal competence for their work.

### 2.2. Data Collection

Data were collected with an instrument called “*the instrument for self-assessment of individual ethos in care of older persons*” and redact ISAEC. Totally, the instrument consists of 58 statements. Twenty-eight statements refer to ethical values as ideals, and 30 statements refer to the possibilities to act in an ethical manner, in the daily work with the older person. A total of 110 questionnaires were handed out by leaders from each unit. Totally, 105 questionnaires were returned, which gives a response rate on 95%.

Total data consist of 6900 observations. Statements in ISAEC instruments were allocated into five groups, as follows: Group I = individual care, Group II = dignified care, Group III = safety care, Group IV = caring communion, and Group V = closeness or/and distance.

The participants were asked to answer the questionnaires by choosing the alternative which best responded to their opinion. The alternatives were stated as follows: not at all agree = 1, partly agree = 2, sometimes = 3, nearly agree = 4, and totally agree = 5 (ethical values as ideals) and never = 1, nearly never = 2, sometimes = 3, mostly = 4, and always = 5 (ethical manners).

These alternatives were textually presented so that the attached numbering was only intended as an index for that particular alternative and not a gradation. In other words, the instrument aims at presenting the alternatives as symbols and not as numerical values. However, as numbers 1–5 were visible in the instrument, it can be expected that the set of alternatives was seen as an ordinal scale, so that, for example, “sometimes” is *before* “mostly,” but there is no distance measure between the two. This is indeed the main reason why we cannot compute with 1–5 as numbers, but rather as symbols in an ordinal scale, and this is why computing with conditional probabilities in Bayesian networks is very suitable.

### 2.3. Data Analysis

#### 2.3.1. Conditional Probabilities and Bayesian Networks

We use Hugin as our tool for Bayesian network generation based on data. Learning from data by Hugin creates a network of nodes connected according to respective conditionalities between nodes. For a higher level of information, clusters of nodes can be created. Clustered nodes are then also linked by conditionalities between nodes.

The structure learning algorithms in Hugin are based on making dependence tests that calculate a test statistic which is asymptotically chi-squared distributed assuming (conditional) independence. If the test statistic is large for a given independence hypothesis, the hypothesis is rejected; otherwise, it is accepted. The probability of rejecting a true independence hypothesis is given by the level of significance, which was selected to be 0.05.

Several methods, including numerical, logical, and probabilistic ones, have been proposed to manage uncertainty in decision-support systems. The probabilistic approach with Bayesian networks are appealing as they capture a computational view of conditional probabilities, which is particularly useful in presence of questionnaires with interdependent questions and using symbolic or ordinal values.

Let *P*(*A*, *B*) be the joint probability for *A* and *B*. Then, the conditional probability is defined as *P*(*A* | *B*) = *P*(*A*, *B*)/*P*(*B*). This then gives the expression *P*(*A*, *B*) = *P*(*A* | *B*)*P*(*B*) for joint probabilities with dependent variables.

The event *A* is said to be *conditionally independent* of event *B* if *P*(*A* | *B*) = *P*(*A*), that is, whenever *P*(*A*, *B*) = *P*(*A*)*P*(*B*).

The previous formulas are used to arrive at Bayes' rule *P*(*A* | *B*) = *P*(*B* | *A*)*P*(*A*)/*P*(*B*) which is the most important rule used and manipulated in Bayesian networks. Bayes' rule makes it possible to calculate conditional probabilities *P*(*A* | *B*), once the opposite conditional probability *P*(*B* | *A*) is known together with the probabilities for the individual events *A* and *B*.

The Bayesian network notation for the probability situation *P*(*A*, *B*) = *P*(*A* | *B*)*P*(*B*) is depicted as


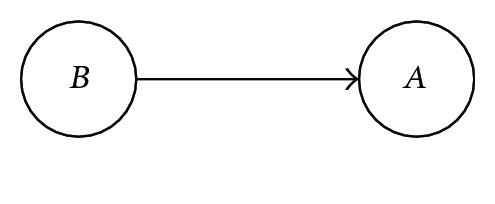


indicating that *B* is conditional to *A*. Similarly, *P*(*A*, *B*) = *P*(*B* | *A*)*P*(*A*) is depicted as


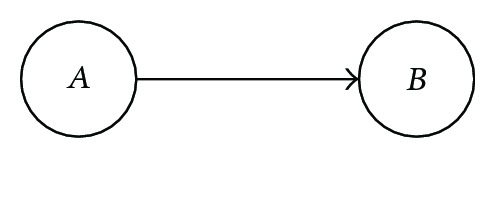


indicating *A* conditional to *B*. Given Bayes' rule, it is then clear that the direction of the arrow is interchangeable depending on the conditional context.

For several nodes, we then need to consider all pairs of conditional probabilities, that is, for the joint probability

(1) $$\begin{array}{c} P\left(A,B,C,D\right)=P\left(A\right)P\left(B\;|\;A\right)P\left(C\;|\;A,B\right)P\left(D\;|\;A,B,C\right). \end{array}$$

We have the depiction


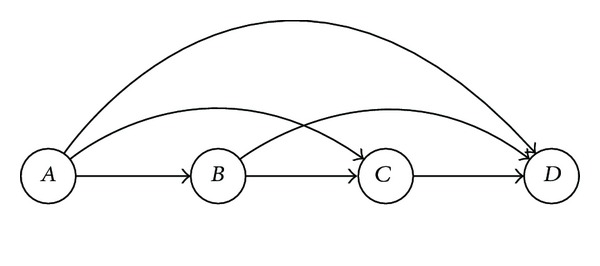


In the most simple cases, events are two valued, and we may, for example, write either *A* = 0 or *A* = 1. A probability like *P*(*C* = 1) is then computed as

(2) P(C=1)=∑a∈{0,1}∑b∈{0,1}∑d∈{0,1}P(A=a,B=b,C=1,D=d),

and a conditional probability like *P*(*A* = 1 | *C* = 1) is computed as

(3) P(A=1 ∣ C=1)=P(A=1,C=1)P(C=1)=∑b∈{0,1}∑d∈{0,1}P(A=1,B=b,C=1,D=d)P(C=1).

In the aforementioned we depict the use of binary data only. Clearly, we can work with more than just two classes of events. In this study, we work mainly with the alternative set {0, 1, 2, 3, 4, 5}, and conditionalities like *P*(*A* ∈ {3,4, 5} | *B* = 4) can be computed for tables in the Results section.

The learning algorithm, as implemented in Hugin, finds the appropriate and correct conditionalities given data. This then creates the Bayesian Belief Network (BBN), where the interconnection defines the structure of the network. This structure and network identification capability is one of the significant advantages of BBN developments. A *cluster* is a subset of entities, so that, on the one hand, no entity in this subset is conditionally dependent with any other entity outside that cluster, and, on the other hand, there are no further subclusters within that cluster. We may also speak of *independent* clusters, to further underline that such clusters with no conditional dependency to their outside world are *molecular*.

### 2.4. Findings

Results are polarized, on the one hand, by *ethical values in the daily care with older people*, and, on the other hand, by *possibilities to act in an ethical manner in the daily care with older people*. However, ethical value and possibility are not complementary or mutually exclusive but rather appear as valuation domains for enabling projection and transformation of given value criteria.

From ethical point of view, good care refers in particular to *dignity*, *participation*, *safety*, *caring community*, and *closeness and distance*, where the latter is concerned mostly with aspects of possibility and the others project to both ethical value and possibilities. This enables, for example, the concept of dignity to be seen as fundamentally ethical at the same time as consideration of dignity becomes a possibility. Similar multimodality with respect to ethical value and possibility can be said about the other concepts, respectively, participation and safety and caring community.

Concepts for ethical value and possibility are further characterized by underlying nodes or entities in clusters related to these concepts, thereby also the entities being members of specific clusters, and conditionalities between these entities.

Among all 58 statements, it turned out that ten statements became one-node clusters; that is, these statements did not show any conditionality with any other statements. These statements were left out of further analysis, since no matter how the dynamics within such a statement is further analysed, it has no effect on any other statement. Indeed, a main objective of this paper is to analyse situations within clusters, where fixation of particular ordinal values for one statement may affect dynamics concerning sibling statements in that cluster. Note that we may speak of sibling nodes, even if it is seldom clear where parent nodes reside in clusters. This is given by the fact that conditionality is really not “directed,” and “changing directions” of conditionalities is done by Bayes' rules.

Structure identification by Hugin also resulted in a number of two-node clusters. Interesting among these clusters were that clashing and enforcing them into one node and restructuring all the remaining nodes provided such two-node clusters to became singleton clusters, in all cases expect for one case where that singleton node become integrated into a larger cluster. This is a motivation for leaving also two-node clusters outside further analysis.

The number of remaining clusters is 9, and the number of nodes in those respective clusters varies from 3 to 7. Among these 9 clusters, 4 clusters contain ethical value statements, and 5 clusters contain possibility statements. The interesting next step is now the semantics of these 9 clusters and their nodes. What is the *name* of the cluster, and what is the *interpretation* of that name? A major part of this Results section is to analyse what happens when certain nodes in clusters are frozen to particular ordinal values. The Bayesian network then recomputes the distribution functions for the other nodes, and the recomputation is enabled by the conditional probabilities. This enables a number of interesting what-if analyses, like if E2, E4, and C2 are fixed at 4, that is, the distribution functions for all these nodes become 100% at ordinal value 4, how does the “move” or “shift” of the function is dynamic for the other nodes?

### 2.5. Ethical Values in the Daily Care with Older People

#### 2.5.1. Cluster I: Dignity

The cluster *dignity* consists of seven nodes or entities. There are small differences between the agreement levels of respective informants. About 85–94 percent of the informants totally agree with all seven statements. In Table 1, we have the distribution function for the cluster *dignity*.

Nodes where level “4” responses in percentage exceed 10 are selected for fixation to a response rate of 100%. These respective fixations are then compared with the shift in response rates for the other nodes, as well as the way they shift in response rates according to the underlying conditional probabilities model of the network.

In order to see this more precisely, let node E2, *to see the needs of the older person*, be fixed at response level “4” = 100%. The influence on node E4, *caring community*, is a 17% shift of level “5” responses to level “4” responses, and on node C2, *encourage the older persons to utilize their own resources*, the effect is an increase of level “4” responses by 18% and level “3” responses by 16%.

Further, for node E4, *caring community*, fixed at level “4” = 100%, node F4, *human love and mercy*, decreases at level “5” by 10% to level “3,” node C2, *encourage the older persons to utilize their own resources*, decreases at level “5” by 10% and at level “4” by 8% with an increase at level “3” by 12% and increase on missing data, that is, “cannot say” data, by 6%, and for A2, *to respect the equal value of each older person*, there is decrease of 13% from level “5,” and as this node had a rather low occurrence of missing data, it is notable how the portion of missing data increases as much as 13% (see Table 2).

Concerning node C2, *encourage the older persons to utilize their own resource*s, changes due to conditionalities for node F4 and E4 are seen in Table 3.

#### 2.5.2. Clusters II–IV

The following clusters are named *community *(cluster II), *safety* (cluster III), and *integrity *(cluster IV). In Table 6, we find the clusters and the named nodes for each cluster. We did not find any dynamical shift in the distribution functions after conditionalities for the nodes had been fixed in level “4” to 100%.

Based on these results, we can state that caregivers, participating in the study, agree that dignity, community, safety, and integrity are important ethical values, in order to guarantee an ethically defensible care, in care with older people.

The last cluster of ethical ideal was named integrity. The distribution function for this cluster is explained in Table 6.

### 2.6. Possibilities to Act in an Ethical Manner in the Daily Care with Older People

Five clusters describe the caregivers' possibilities to act in an ethical manner, within their daily work with the older persons. The clusters are named as follows: *possibility for closeness and distance, community, dignity, safety, *and* participation*. The degree of coherence between the statements and the own opinions varies significantly between the informants.

A dynamical shift in the distribution functions, when conditionalities for respective node have been changed, can be found within all clusters. It is apparent that the shape of the clusters is affected as the underlying conditions and properties change, for example, with respect to change of caregivers, as new personnel enter the unit, and the condition spectra change at the unit either for particular individuals or by individuals leaving and new patients entering. The changes obviously affect care intensity as a whole. Further, organizational and administrative aspects and/or changes may also have effect on ethical manner.

#### 2.6.1. Cluster V: Possibility for Closeness and Distance

Cluster V consists of three nodes: *sensitivity, professional approach,* and* a genuine interest in the quality of life of the older person*. The nodes are closely intertwined, and dynamics of the conditionality in respective nodes affects each other within the cluster. Fixation in one node implies adjustments of frequencies in the other nodes. Thereby changes like fixations at level “4” will imply downshifts in the other nodes towards levels “4” and “3.” Thereby, ethical manners, as caregivers' *professional approach *and *interest for the older person, *will be important entities for upholding closeness and/or distance in the daily work with the older person (Table 7).

#### 2.6.2. Cluster VI: Possibilities for Dignified Care

Cluster VI consists of six nodes (the nodes and the distribution function for the cluster are presented in Table 8).

For upholding dignified care, entities as *respecting the philosophy of life, creating a meaningful life,* and *continuously keeping the older patients informed on the phenomena of significance* for their health and wellbeing, will be of most importance. As many as 18 percent feel that their possibilities to continuously inform the older about their health situation remain at level “3,” and 10 percent of the informants state that they *“sometimes”* have possibilities to respect *the philosophy of life* of the older person. About 16 percent of the informants feel they have limited possibilities to create a meaningful life for the older patients.

We shall see what most likely happens inside the cluster if we fix some of the entities at level “4” to be 100 percent. A dignified care seems to be dependent on caregivers' attitude to the older patients. A fixation at level “4” for node B21 (*treating the older as an adult person*) will imply downshifts in nodes F21 (*to continuously inform the older person*), C21 (*to respect the philosophy of the life of the older*), and E21 (*to create a meaningful life for the older*).

The following example also reinforces the attitude of the caregivers as an important factor for opportunities to enable dignified care for the older persons. A fixation at level “4” for node E21 (*to create a meaningful life for the older*) downshifted nodes D21, A21, and B21, at level “3” about 4–19 percent (Table 10).

These two examples show how the attitude of caregivers makes distinctions about upholding the dignity of the older person in the daily work, and we can see the same tendencies with fixation at level “3” or “4” for the other nodes within the cluster.

#### 2.6.3. Cluster VII: Possibilities for Participation

The cluster consists of seven nodes, where the nodes including *freedom* and *believing* the older person are entities that informants perceive as important entities to facilitate patient involvement in their own care. The distribution functions show that *ability for the patients to participate* in their own care is limited. The *caregivers' desire to care for the same patient during a longer period, sharing moods with the older*, and *being humble when facing the older person* provide further dimensions for evaluations and enrichment as related to patients themselves given possibilities for participation. Making use of the older person's resources and capacities is a real challenge, and on the basis of the results, it appears that caring is more about doing *for* than doing *with* the patient, that is, indeed being participative in these respects.

What happens within the cluster if a fixation at level “4” to 100% is done for some of the nodes in the cluster? Nodes A41 (*promote continuity and preservation in the care process as a whole*), F41 (*being emphatic and sharing the moods with the older person*), A11 (*support resources of the older patient*), G41 (*being humble while caring for the older person*), and D41 (*encourage participation*) being affected both upwards and downwards in the set of alternatives, as well as participation of the patient, are dependent on caregiver attitudes. The effects on distributions for respective nodes are described in Tables 11, 12, 13, and 14.

The node with the strongest effect on the nodes within the cluster is D41. The way caregivers feel about *encourage participation *has effect on each of the nodes within Table 12.

#### 2.6.4. Cluster VIII: Possibilities for Safety Care

Cluster VIII consists of three nodes, respectively, to *be responsible for the inner safety of older persons, to create a safe situation for the older person,* and *protect against mistreatment of the older person* (see Table 16).

#### 2.6.5. Cluster IX: Possibilities to Create a Caring Communion

The final cluster, *caring communion*, contains seven nodes, which are described in Table 15. A caring communion will be established when the older person is in charge of his/her own life. In these situations, caregivers really want to fulfil the needs of the older person and thereby also show respect for the older person, that is, *show respect for the older person, be flexible, *and *encourage participation* (Table 17).

The potential for a caring communion to reach care relationships is dependent on the *desire to listen *to the older person, *monitoring of older person's satisfaction about care*, *flexibility,* and *respect for the older person's own decisions*. Node C11 (flexibility) has the greatest ability to affect other nodes within the cluster. Fixing node C11 level to “4,” corresponding to an improvement of the original result, the number of observations for each node increases (Table 18).

## 3. Discussion

In the study of ethical values and ethical manners, the Bayesian approach, represented by the use of Bayesian Belief Networks (BBNs), has been a useful method, because of the ability to compute with symbolic data. In particular, the ability to show the effects of using extended conditionalities, involving both original nodes as well as clusters of nodes, has been a useful insight. The computing process, as enabled by the structure identification capability of BBNs, generated clusters of network structures and ended up in a total of nine clusters. Four clusters described ethical values (ethos), and five clusters explained possibilities as experienced by caregivers and how to act in an ethical manner in the daily work with the older people.

Clusters consist of a BBN of nodes (three to seven nodes per cluster), and the relation between nodes within a cluster forms the specific character of the cluster. The comprehension of clusters is therefore given by the statements, in form of conditional probabilities, within that specific BNN. We can state that the Bayesian approach had possibilities to generate clusters and underlying structural entities of relevance for the aim of the study. The structure identification capabilities enabled to find distinct dynamics within the clusters, as soon as the conditions of individual nodes were changed from the initial conditions.

Our intension was to find “what-if questions,” like *“what happens if the conditions of one or several specified nodes within a cluster are changed?,”* in order to prevent irregularities in the area of ethics. The dynamics of fixation of one or more entities to a given level can be seen in the cluster *dignity* consists of seven nodes or entities. There are small differences between the agreement levels of respective informants. About 85–94 percent of the informants totally agree with all seven statements. In Table 1, we have the distribution function for the cluster *dignity*.

Tables 1, 4, 5, and 6 represent changes between different assessment levels. We have to note that the material for the study was limited, and far-reaching conclusions cannot be made, but the results of the study point at entities possess power to change ethical ideals and manners in a remarkable way. This insight is of vital importance for the development of ethical manners in daily nursing care. Ethical values (ideals) such as integrity, dignity, safety, and caring community are embraced by the majority of respondents. However, we still have to note that cluster *dignity* has three percent (*n* = 735) of the observations at level “3” (sometimes) or lower, while within cluster *integrity* four percent (*n* = 315). Within this group of clusters, *dignity* was the only cluster which showed significant effects when the conditions for any of the nodes were changed.

Clusters describing ethical manners were the following: *closeness, distance, dignity, safety, participation,* and *caring communion*. Within these clusters, we can see numbers of significant effects if the conditionalities for given node were changed (Tables 9, 10, 12, and 15). Powerful negative effects on the entities probably change the opportunities for the older person to have good ethical care. However, earlier studies highlight the importance to develop an ethical culture at the unit. Without ethical discussions and models, the way to act in ethically critical situations, each caregiver acts in their own way, and it will not be possible to guarantee the older person ethical good care, because the quality of the care is depending on the individual caregiver's attitudes and manners [27, 28]. Promoting ethical good care is the responsibility of the whole work team.

Caregivers participating in the study indeed approved ethos as it was expressed in the clusters of dignity, community, security, and integrity. A certain dynamics can be seen within cluster dignity, that is, coherence between the statement and the opinion of the informant. This was clear particularly in relation to basic care needs and in care situations with older people who generally suffer from frailty and increasing degree of cognitive decline.

The study shows that the caregivers' attitudes to entities like compassion and mercy, moments of calm, respect, and compliance with the wishes and needs of the older person are of major importance in maintaining a dignified care. The perception and comprehension of the older person, as a person who lived a rich and meaningful life and where disease and illness changed that person's life, is important for the formation of ethical manner. Positive approaches to the older person, seeing the person behind the illness and suffering, are basic prerequisites for ethical manners in the daily care. Based on earlier research and results from this study, we know that the view of the older person and knowledge about ageing processes are some of the most important entities in the daily care of older persons [29–31]. Respect and dignity of the older person were also in earlier research proven to constitute one of the major caring challenges. The views of the older person link to numerous ethical challenges, and therefore greater attention should be considered in both education and training of caregivers.

Tornstam shows in his theory of gerotranscendence how the ageing process results in a value of displacement in the elderly [29, 32]. The older persons experience about their life as meaningful, having the right to control their lives regardless of the health and functional status, and feeling respected is important entity for the experience of dignity. Wadensten and Carlsson [31, 33, 34] showed in their studies of the previous phenomena, that the caregivers did not properly perceive and comprehend the older person. In order to guarantee ethical care for the elderly, it seems likely that the view of the elderly needs to be changed, and the elderly should be seen as an adult with her own life to live, regardless of health or illness. Wadensten and Carlsson [31, 33] state that caregivers have not observed the value shifts as Tornstam [29] explained in his study.

Earlier research and findings [8–10, 12, 20] from this study are consistent with each other. Opportunities for unethical manners and ethical challenges are in previous research described in a rather context dependent and descriptive way, and often without prospective and prognostic aspects. This study aims to include foresightedness and to highlight the probable effects on and prospects of ethical care. If we do not take these negative attitudes seriously, as we can see in the findings section, good ethical care will be at risk.

On the basis of the present study, we can only state that there are differences in caregivers attitudes. Some caregivers felt it was “almost” or “every time” possible to act in an ethical manner, while others did not feel they had sufficient possibilities to act ethically. The reasons for those possibilities being limited is not confirmed in the present study but appear in some previous research [13, 35, 36]. In addition to the views of the elderly and knowledge about the ageing process, we should further include observations about situations where the total resources are not in balance to meet the needs of the elderly [37, 38]. Different opinions within the care unit about the caregivers ability to provide care in accordance with the ideals, related to good ethical care, moral anxiety, and guilt, are further circumstances [36] to be considered beyond the scope of this study.

This stress is clearly related to the quality of care. The situation can become very serious. Earlier research reports about serious medical errors and even death, illness, indifference, and arrogance [35–37].

We cannot overlook leader's responsibilities, as leadership sets the norms and upholds a culture [39]. The leaders support and create conditions for caregivers to act in accordance with the collective agreements about ethical good care, but they also explicitly provide and disseminate the ethical care criteria for and within the organisation. Leaders also take questions about resource allocation seriously, by creating human and material conditions for caregivers to act in accordance with their ethical ideals [39].

This study is unique in its kind, both concerning the selected approach and methodological questions. BBNs have not been used significantly in nursing research, nor are there any studies that examine the ethical possibilities with focus on the probable effects upon changing conditions.

An important point to make is that results must be understood given the translation of symbolic data from numbers to logical concepts and statements. Ethical ideals and ethical manners are phenomena that indeed make no sense to be explained exclusively by numeric data. The data represented and presented in the study are seen as symbolic data and appear, for example, as clusters of ethical ideals and attitudes which obtain their special character of the entities (nodes) and forms the network within the cluster. The character of a given cluster depends on the explanation given the cluster. The nine clusters within the study are interpreted from caring science perspective [7, 24–26] and earlier research about ethical questions in the daily work with the older person. In view of this transformation from numeric to statements, we have thus moved along the path *from statistics to logic*.

## 4. Conclusion

The study has opened up new opportunities to prevent services from becoming increasingly impersonal and stereotypical, and instead becoming care-based concerning ethos with ethical manners.

The nodes that describe the cluster's character may in the future not only serve as a basis for ethical discussions and decision-making, but also be starting points for caregiver's individual development.

The structure of the cluster with the underlying entities, that the study generated, seems to be an interesting development of the ISAEC instrument. The clusters with underlying nodes could be used as a framework for the continued development of instruments to identify caregiver attitudes to ethical values and ethical approach.

In summary, we can state that the study has enriched the ethical discussion and opened up new ‘‘what-if” questions. That in turn creates awareness of barriers to ethical good care.

## Figures and Tables

**Table 1:** Cluster I: Dignity.

		Distribution function for Cluster I (percent)					
		5	4	3	2	1	Missing
*F4 *	Human love and mercy	*88,2 *	*9 *	*2,9 *			
C5	Moments of peace	94,3	5,7				
E2	To see the needs of the older person	85,7	10,5	1			2,9
B1	Respect the needs and wishes of the older person	87,6	8,6	1,9			1,9
E4	Caring community	87,1	12,4	0,5			
C2	Encourage the older persons to utilize their own resources	85,7	10,5	2,9			1
A2	To respect the equal value of each older person	94,3	2,9	1			1,9

**Table 2:** Effects on distribution function when E4 fixed to “4” = 100%.

		5	4	3	2	1	Missing
F4	Human love and mercy	−11,51	1,91	9,59			
C2	Encourage the older persons to utilize their own resources	−10,5	−8,06	12,1			6,46
A2	To respect the equal value of each older person	−19,3	4,84	1,35			13,11

**Table 3:** Effects on distribution function when C2 fixed to “4” = 100%.

		5	4	3	2	1	Missing
F4	Human love and mercy	−9,03	11,32	−2,3			
E4	Caring community	8,15	−9,5	1,36			

**Table 4:** Cluster II: Community.

		Distribution function for Cluster II					
		(percent)					
		5	4	3	2	1	Missing
A4	Caring community	94,3	5,7				
B3	Listen to the wishes of the older person	85,7	10,5	1			2,9

**Table 5:** Cluster III: Safety.

		Distribution function for Cluster III					
		(percent)					
		5	4	3	2	1	Missing
A3	The experience of confidence	99					
E3	Professionally caregivers make safety	96	3		1		
D3	Knowledge make safety	79	13				2

**Table 6:** Cluster IV: integrity.

		Distribution function for Cluster IV (percent)					
		5	4	3	2	1	Missing
D1	The older person is unique with unique needs	84	13	1			2
B4	Inviolability	88	9	3			
C3	Be responsible for the safety of the older person	89	6	2	2		1
C4	Respectfully done nursing	95	4	1			

**Table 7:** Cluster V: Possibilities for closeness and distance.

		Distribution function for Cluster V (percent)					
		5	4	3	2	1	Missing
B51	Sensitivity	48	39	8	3		2
E51	Professional approach	47	38	10	2		3
C51	A genuine interest in the quality of life of the older person	34	45	14	3		4

**Table 8:** Cluster VI: Possibilities for dignified care.

		Distribution function for Cluster VI (percent)					
		1	2	3	4	5	Missing
C21	Respecting the philosophy of live of the older person	52,4	31,4	10,5	1,9	1	2,9
A21	Observing the older person's dignity	55,2	39,1	3,8	1,9		
B21	Treating the older person as an adult person	63,1	32,2	3,7			1
E21	Creating a meaningful life for the older person	41,9	41,9	10,5		1	4,8
F21	Continuously informing the older person	27,6	44,8	18,1	3,8		5,7

**Table 9:** Effects on distribution function when B21 fixed to “4” = 100%.

		5	4	13	2	1	Missing
D21	The older person is treated respectfully regardless of health status	−2,2	3,21	0,28			−1,29
C21	Respecting the philosophy of life of the older person	−6,53	8,63	0,63	2,05	−0,26	−2,61
A21	Observing the older person's dignity	−5,2	6,14	0,5			−1,44
E21	Creating a meaningful life for the older person	−9,93	11,4	2,55		0,53	−3,6
F21	Continuously informing the older person	−16,06	24,67	−5,03	1,62		−5,2

**Table 10:** Effects on distribution function when E21 fixed to “4” = 100%.

		5	4	3	2	1	Missing
D21	The older person is treated respectfully regardless of health	−20,91	15,54	4,47			
A21	Observing the older person's dignity	−36,37	20,45	15,91			
B21	Treating the older person as an adult person	−36,46	15,51	18,57			

**Table 11:** Cluster VII: Possibilities for participation.

		Distribution function for Cluster VII (percent)					
		5	4	3	2	1	Missing
C1	Freedom for the older person	77,14	18,1	1,9			2,86
E1	To believe in the older person	89,52	8,57	0,95			0,95
D41	Encourage participation	33,43	42,65	14,45	6,09		3,38
A41	Promote continuity and preservation in the care process as a whole	23,81	42,86	24,76	3,81	0,95	3,81
F41	Being emphatic and sharing the moods with the older person	29,5	40	24,76	2,86		2,86
A11	Support resources of the older person	15,24	57,14	24,76	1,9		0,95
G41	Being humble while caring for the older person	42,8	36,84	13,52	5,36		1,47

**Table 12:** Effects on distribution function when D41 fixed to “3” = 100%.

		5	4	3	2	1	Missing
C1	Freedom for the older person	−4,4	6,36	−1,14			−0,82
E1	To believe in the older person	−0,48	1,33	−0,6			−0,27
A41	Promote continuity and preservation in the care process	−2,61	13,24	−7,06	−0,57	−0,52	−2,48
F41	Being emphatic and sharing the moods with the older person	−10,87	6,27	7,99	−1,79		−1,57
A11	Support resources of the older person	−3,31	−0,17	2,87	0,46		0,15
G41	Being humble while caring for the older person	−1,9	−2,43	3,61	1,57		−0,83

**Table 13:** Effects on distribution function when A41 fixed to “4” = 100%.

		5	4	3	2	1	Missing
D41	Encourage participation	−6,8	13,18	−2,87	−2,16		−1,36
G41	Being humble while caring for the older person	5,26	6,57	−10.89	−0,04		−0,9

**Table 14:** Effects on distribution function when F41 fixed to “4” = 100%.

		5	4	3	2	1	Missing
D41	Encourage participation	−2,73	6,68	1,68	−4,18		−1,47
A11	Support resources of the older person	−10,48	9,53	−0,95	0,48		1,43
G41	Being humble while caring for the older person	6,08	5,55	−6,69	−4,41		−0,52

**Table 15:** Effects on distribution function when A11 fixed to “4” = 100%.

		5	4	3	2	1	Missing
D41	Encourage participation	−0,65	−0,13	0,31	0,04		0,42
F41	Being emphatic and sharing the moods with the older	−4,5	6,67	−4,76	0,47		2,14
G41	Being humble while caring for the older person	0,12	0,48	−0,86	−0,26		0,54

**Table 16:** Cluster VIII: possibilities for safety care.

		Distribution function for Cluster VIII (percent)					
		5	4	3	2	1	Missing
D31	Be responsible for the inner safety of older person	66,7	26,6	3,8			2,9
A31	To create a safe situation for the older person	30,0	47,0	17,0	3,0		3,0
B31	Protect against mistreatment of the older person	51,0	26,7	13,0	4,0	0,6	4,7

**Table 17:** Cluster IX: possibilities for caring communion.

		Distribution function for Cluster IX (percent)					
		5	4	3	2	1	Missing
B11	Person-centred care	30,2	49,7	17,7			2,4
E41	The desire to listen to the older person	41,9	44,8	9,5			3,8
D11	Monitoring of older person's satisfaction about care	56,2	29,5	11,4	0,9		1,9
B41	True presences	44,8	42,9	9,5			2,9
C11	Flexibility	4,6	45,9	41,7	6,5		1,5
C41	Encourage participation	41	41,9	13,3	1		2,9
F/E11	Respect for the older person's own decisions	3,8	13,3	34,3	29,5	10,5	8,6

**Table 18:** Effects on distribution function when C11 “4” = 100%.

		5	4	3	2	1	Missing
B11	Person-centred care	5,57	−0,57	−3,48			−1,52
E41	Listen to the older person	2,21	−1,15	−1,25			4,01
D11	Monitoring of older person's satisfaction about care	14,17	−3,01	−10,58	−0,55		
B41	True presences	5,09	0,25	−5,82			1,47
C41	Encourage participation	−1,54	0,5	2,03	−0,05		−0,92
F/E11	Respect for the older person's own decisions	−3,55	−6,64	1,7	9,32	4,13	−4,97
